# The association between cigarette smoking exposure and sarcopenia assessed by Psoas Muscle Index: A secondary data analysis

**DOI:** 10.18332/tid/222553

**Published:** 2026-07-26

**Authors:** Osman Onur Sarı, Hasan Sözel, Yıldız Kılar Sözel

**Affiliations:** 1 Department of Internal Medicine, Akdeniz University Hospital, Antalya, Turkey; 2 Department of Radiology, Antalya Education and Research Hospital, Antalya, Turkey

**Keywords:** smoking, sarcopenia, Psoas Muscle Index, computed tomography, muscle mass

## Abstract

**INTRODUCTION:**

Sarcopenia is a geriatric syndrome characterized by a progressive loss of skeletal muscle mass and strength, leading to decreased physical capacity, impaired quality of life, and increased mortality. Although smoking has multiple systemic effects, its relationship with sarcopenia remains controversial. This study aimed to investigate the relationship between varying levels of cigarette smoking exposure and sarcopenia, assessed using the Psoas Muscle Index (PMI) measured using non-contrast abdominal computed tomography (CT).

**METHODS:**

This cross-sectional analysis included 261 adult patients who underwent non-contrast abdominal CT between 2019 and 2022 at Akdeniz University Internal Medicine and Urology outpatient clinics. The patients were categorized into three groups: active smokers, passive smokers, and non-smokers. Passive smokers were defined as non-smoking individuals who reported regular exposure to secondhand cigarette smoke for at least two days per week. Individuals with conditions or medications known to affect body composition were excluded from the study. Bilateral psoas muscle cross-sectional areas at the L3 vertebral level were manually measured, and PMI was calculated (cm^2^/m^2^). Sarcopenia was defined based on sex-specific PMI cutoff values derived from the lowest quintile (20th percentile) of the study population (males: ≤4.421 cm^2^/m^2^; females: ≤3.150 cm^2^/m^2^). Statistical analyses were performed using Kruskal-Wallis and Mann-Whitney U tests for continuous variables, chi-squared test for categorical variables, and multivariate linear and logistic regression models to adjust for potential confounders.

**RESULTS:**

There were no statistically significant differences in PMI values among active, passive, and non-smokers (p=0.138). Laboratory parameters, including C-reactive protein (CRP), albumin, alanine aminotransferase (ALT), aspartate aminotransferase (AST), and glomerular filtration rate (GFR), were also comparable between the groups. Hemoglobin levels were significantly higher in active smokers compared to non-smokers (active: 14.40 [IQR: 13.00–15.25] vs non-smoker: 13.50 (IQR: 12.90–14.60) g/dL; p=0.029). Sarcopenia prevalence did not differ significantly among the three groups (non-smoker: 16.1%, passive smoker: 28.7%, active smoker: 16.1%; p=0.057).

**CONCLUSIONS:**

In this cohort, neither passive nor active cigarette smoking exposure was associated with significant changes in Psoas Muscle Index or sarcopenia prevalence. Although smoking is a well-established risk factor for many diseases, its direct effect on muscle mass remains inconclusive. Further prospective studies considering smoking intensity, duration, and other confounders are warranted to clarify this relationship.

## INTRODUCTION

Sarcopenia is a progressive syndrome characterized by a decline in the overall skeletal muscle mass and strength. It is associated with reduced physical capacity, a poor quality of life, and increased mortality. As the global elderly population continues to increase, the significance of sarcopenia as a public health issue has become increasingly prominent.

Previous studies have reported varying prevalence rates of sarcopenia. Many studies have indicated that sarcopenia affects 5–25% of individuals aged 60–70 years and 11–50% of those aged >80 years. One study identified a 6% loss in muscle mass every decade after the age of 45 years^1^. According to a study conducted in Turkey using the European Working Group on Sarcopenia in Older People (EWGSOP) criteria, the prevalence of sarcopenia in the 40–49, 50–59, 60–69, 70–79, and 80 years age groups was 7%, 10.6%, 15.4%, 21.2%, and 36.5%, respectively^2^. In the IL-SIRENTE study conducted in Italy by Landi et al.^3^, the prevalence was 25.7% in men and 19.8% in women.

Differences in measurement techniques and reference values used in diagnosing sarcopenia, along with variations in age, sex, ethnicity, and living environment (hospital, nursing home, home), contribute to the disparity in prevalence. The pathophysiological mechanisms proposed to underlie sarcopenia include muscle remodeling, apoptosis of muscle cells, alterations in muscle protein turnover, and loss of motor neurons. However, the specific effects of these mechanisms on muscle mass and strength remain unclear. Genetic predisposition has also been proposed to explain the inter-individual and intergroup variations in sarcopenia prevalence^4^. Other contributing factors include sedentary lifestyle, malnutrition, endocrine disorders, impaired tissue repair, chronic diseases, and malignancies^5^.

Sarcopenia has been identified as a negative prognostic factor in several conditions, including malignancy, cirrhosis, chronic kidney disease, coronary artery disease, and heart failure, with significantly higher morbidity and mortality observed in sarcopenic patients across these disease states^6-14^. Smoking is one of the leading modifiable risk factors for disease and mortality. The biological mechanisms by which cigarette smoking may contribute to muscle loss are multifaceted. Tobacco smoke contains oxidative compounds that promote systemic inflammation and increase reactive oxygen species (ROS), both of which accelerate protein degradation and impair muscle protein synthesis. Nicotine has been shown to suppress insulin-like growth factor-1 (IGF-1) signaling, a key anabolic pathway for skeletal muscle maintenance. Furthermore, chronic smoking is associated with elevated levels of pro-inflammatory cytokines such as TNF-α and IL-6, which activate the ubiquitin-proteasome pathway and contribute to muscle atrophy. Smoking-induced hypoxia may further impair mitochondrial function and reduce the oxidative capacity of skeletal muscle fibers^15^. Although many studies have investigated the detrimental health effects of smoking, studies on its relationship with sarcopenia have yielded conflicting results. Some studies have found a significant association, while others have not. A 2014 meta-analysis concluded that smoking may only have a minimal effect on sarcopenia, emphasizing the need for further research^16^. Several longitudinal cohort studies have demonstrated that smoking is independently associated with accelerated muscle mass decline over time, and that cumulative smoking exposure measured in pack-years is independently associated with lower skeletal muscle mass index^17,18^. These findings suggest a temporal and potentially dose-dependent relationship between tobacco exposure and skeletal muscle deterioration.

Magnetic resonance imaging (MRI) and computed tomography (CT) are considered the gold standard methods for evaluating skeletal muscle mass and diagnosing sarcopenia. In retrospective studies utilizing existing clinical data, CT-derived PMI represents the most feasible and methodologically rigorous measure of muscle mass available. Sarcopenia can be assessed by measuring the cross-sectional area of the psoas muscle in the third or fourth lumbar vertebrae (L3 or L4). The Psoas Muscle Index (PMI) is calculated by dividing the muscle area (in cm^2^) by the square of the height (in m^2^)^19^.

Among the modifiable lifestyle factors potentially influencing skeletal muscle mass, cigarette smoking has emerged as a particularly relevant yet understudied risk factor. While the systemic effects of smoking are well-documented, the specific relationship between cigarette smoking – particularly passive smoking – and CT-derived psoas muscle mass in otherwise healthy adults remains poorly characterized. Most existing studies have focused on diseased or elderly populations, and few have examined passive smoking as an independent exposure variable using objective imaging-based muscle assessment. We aimed to address this gap by objectively evaluating the association between active and passive smoking exposure and CT-defined low psoas muscle mass using CT-derived PMI at Akdeniz University Hospital, Antalya, Turkey.

## METHODS

### Study design and population

In this cross-sectional analysis of secondary data, 261 patients who underwent non-contrast abdominal CT between 2019 and 2022 at Akdeniz University Hospital, a tertiary care academic medical center in Antalya, Turkey, were identified from the hospital database. Data were retrospectively collected from electronic medical records and the radiology archive by O.O.S. Patient information was retrieved through a review of physical and electronic medical records.

**Figure 1:**
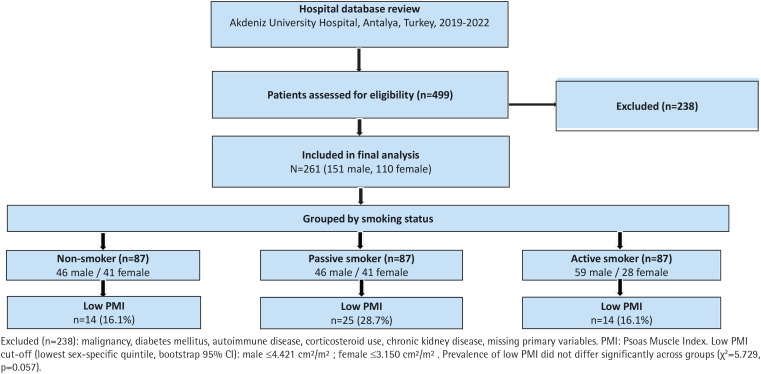
Participant selection flow diagram

Inclusion criteria were: 1) age ≥18 years, 2) availability of abdominal CT imaging at the L3 vertebral level; and 3) documented smoking status in the medical records. A flow diagram illustrating the participant selection process is presented in Figure 1. Patients were excluded if they met any of the following criteria: age <18 years, history of active infection, diagnosis of malignancy, diagnosis of diabetes mellitus, chronic inflammatory rheumatologic or autoimmune disease, pregnancy, use of medications that could affect body composition (e.g. diuretics or corticosteroids), or medications that could alter thyroid function tests (e.g. beta-blockers and amiodarone).

### Smoking status evaluation

Smoking status and passive smoking exposure were assessed via structured self-report at the time of clinical interview. Patients were categorized into three groups: active smokers (individuals who reported currently smoking cigarettes), passive smokers (non-smoking individuals who reported regular exposure to secondhand cigarette smoke for at least two days per week, consistent with definitions used in previous epidemiological studies), and non-smokers (individuals who had never smoked and reported no secondhand smoke exposure)^20^. The smoking duration and pack-year data of patients could not be retrieved because this information was not systematically recorded in the existing medical records at the time of data collection.

### Imaging analysis

Non-contrast abdominal CT images were used to evaluate the cross-sectional areas of the psoas muscle. Total Psoas Area (TPA) was defined as the combined cross-sectional area of the bilateral psoas muscles in cm^2^ measured at the third lumbar vertebra (L3) level. The Psoas Muscle Index (PMI) was subsequently calculated as TPA divided by the square of each patient’s height in meters (TPA/height^2^, cm^2^/m^2^). A radiologist with 7 years of experience in body composition assessment manually delineated the bilateral psoas muscle areas at the mid-level of the third lumbar vertebra using a dedicated imaging software program (Sectra Workstation IDS7, Version 24.2.16.6066 ©2023 Sectra AB).

To determine sarcopenic status, sex-specific PMI cutoff values were established using the lowest quintile (20th percentile) of PMI within the study cohort, a well-established approach in CT-based body composition literature^19^. This population-specific approach was preferred over generic international thresholds, as CT-derived PMI cutoffs have not been universally standardized across ethnic populations, and applying thresholds derived from Western or Asian cohorts to a Turkish population may introduce significant misclassification bias. The resulting sex-specific cutoff values were 4.421 cm^2^/m^2^ (95% CI: 4.179–4.621) for males and 3.150 cm^2^/m^2^ (95% CI: 3.082–3.261) for females, derived via bootstrap resampling with 1000 iterations. Participants with PMI at or below these thresholds were classified as having CT-defined low psoas muscle mass. It is acknowledged that these cutoffs were not validated against EWGSOP2 or AWGS2 criteria, which also require assessment of muscle strength and physical performance – measurements unavailable in this retrospective dataset.

### Ethical approval

The study was conducted in accordance with the Declaration of Helsinki and approved by the Ethics Committee of Akdeniz University (Date: [13/09/2023], Decision No: [KAEK-710]). Due to the retrospective nature of the study, the requirement for informed consent was waived by the ethics committee.

### Data analysis

Data were analyzed using IBM SPSS Statistics 25, © Copyright SPSS Inc. Participants with missing data for any primary variable (PMI, smoking status, height, weight, age, or sex) were excluded from the analysis; no imputation was performed. Continuous variables are presented as mean ± standard deviation (SD) for normally distributed data and as median with interquartile range (IQR) for non-normally distributed data. Categorical variables are presented as frequencies and percentages. The conformity of continuous variables to a normal distribution was analyzed using Kolmogorov-Smirnov and Shapiro-Wilk tests, with the appropriate test selected depending on the number of samples. In independent two-group analyses, an Independent Samples t-test was employed for datasets exhibiting a normal distribution, while a Mann-Whitney U Test was utilized for those failing to demonstrate normal distribution. In cases where there were more than two groups and the data were normally distributed, one-way ANOVA was employed. Conversely, when the data were not normally distributed, the Kruskal-Wallis H test was used. When the one-way ANOVA yielded statistically significant results, the *post hoc* Scheffe test was employed to ascertain the source of the discrepancy. In accordance with the outcome of the Kruskal-Wallis H test, a *post hoc* Bonferroni correction was conducted. In the correlation analysis between continuous variables, a Spearman’s rho correlation analysis was conducted because of the non-normal distribution of the data. In this study, a statistical significance level of 0.05 was deemed acceptable. PMI was found to be non-normally distributed and was therefore analyzed using the Kruskal-Wallis test with *post hoc* Mann-Whitney U and Bonferroni correction. To assess the independent association between smoking status and PMI, multivariate linear regression was performed with PMI as the continuous dependent variable, adjusting for BMI, sex, and age. Additionally, multivariate logistic regression was performed with CT-defined low psoas muscle mass (yes, no) as the binary dependent variable, adjusting for the same covariates. Results of logistic regression are reported as odds ratios (OR) with 95% confidence intervals (CI) and p values.

## RESULTS

A total of 261 participants were included in this analysis (151 males, 110 females; mean age 44.7 ± 9.9 years, range 25–76). Participants were equally distributed across three smoking groups: non-smokers (n=87), passive smokers (n=87), and active smokers (n=87). Overall, 53 participants (20.3%) were classified as having CT-defined low psoas muscle mass based on sex-specific PMI cutoff values (males: ≤4.421 cm^2^/m^2^; females: ≤3.150 cm^2^/m^2^). The prevalence of CT-defined low psoas muscle mass did not differ significantly across smoking groups (non-smoker: 14/87 [16.1%]; passive smoker: 25/87 [28.7%]; active smoker: 14/87 [16.1%]; χ^2^=5.729, p=0.057), though a numerically higher prevalence was observed in passive smokers. The age distribution did not differ significantly across groups (non-smoker: 44.0 [IQR: 38.0–52.0]; passive: 40.0 (IQR: 36.5–51.5); active: 45.0 [IQR: 39.0–51.0] years; p=0.150). Sex distribution also did not differ significantly across groups (non-smoker: 46M/41F; passive: 46M/41F; active: 59M/28F; χ^2^=5.311, p=0.070). Mean height differed significantly across groups (non-smoker: 167.0 ± 8.9 cm; passive smoker: 168.1 ± 7.5 cm; active smoker: 170.1 ± 7.5 cm; p=0.031), with active smokers being significantly taller than non-smokers on *post hoc* analysis (p=0.012). Body weight did not differ significantly across groups (non-smoker: 76.0 [IQR: 70.0–85.0]; passive: 75.0 (IQR: 67.0–81.0); active: 77.0 (IQR: 67.0–85.0) kg; p=0.367). BMI was significantly higher in non-smokers compared to both passive and active smokers (non-smoker: 27.36 (IQR: 24.91–30.88); passive smoker: 26.08 (IQR: 24.02–28.14); active smoker: 26.15 (IQR: 23.84–28.54) kg/m^2^; p=0.017). BMI did not differ significantly between the passive and active smoker groups (Table 1).

**Table 1:** Demographic and clinical characteristics of study participants by smoking status, Akdeniz University Hospital, Antalya, Turkey, 2019–2022 (N=261)

*Characteristics*	*Passive smokers*	*Active smokers*	*Non-smokers*	*Total*	*p*
Age (year)	40 (36–52)	45 (39–51)	44 (38–52)	44.7 ± 9.9	0.150^a^
Height (cm)	168.1 ± 7.5	170.1 ± 7.5	167.0 ± 8.9	168.5 ± 8.2	**0.031^b^**
Weight (kg)	75 (67–82)	77 (67–85)	76 (70–85)	77.8 ± 15.4	0.367^a^
BMI (kg/m^2^)	26.08 (24.02–28.14)	26.15 (23.8–28.54)	27.36 (24.91–30.88)	26.42 (24.02–29.00)	**0.017^a^**
**Gender,** n (%)					0.070*
Female	41 (47.1)	28 (32.2)	41 (47.1)	110 (42)	
Male	46 (52.9)	59 (67.8)	46 (52.9)	151 (58)	

Values presented as mean ± SD or median (IQR). P-values refer to between-group comparisons. Statistical significance threshold: p<0.05.

a Kruskal-Wallis H Test, *post hoc* Bonferroni correction.

b One-way ANOVA, Scheffe *post hoc* test.

* Pearson chi-squared test, Yates correction.

There were no statistically significant differences in albumin (p=0.408), C-reactive protein (p=0.285), alanine aminotransferase (p=0.435), aspartate aminotransferase (p=0.772), or glomerular filtration rate (p=0.523) values between the three groups. Hemoglobin levels were significantly higher in active smokers compared to both non-smokers and passive smokers (active: 14.40 (IQR: 13.00–15.25) g/dL; non-smoker: 13.50 (IQR: 12.90–14.60) g/dL; passive: 13.70 (IQR: 12.80–14.70) g/dL; p=0.029). No significant difference was observed between non-smokers and passive smokers. This finding is consistent with smoking-induced secondary polycythemia and is noted as a secondary observation not directly related to the primary research question.

Total Psoas Area (TPA), the combined cross-sectional area of bilateral psoas muscles at L3, did not differ significantly across smoking groups (non-smoker: 1368.6 ± 464.5 cm^2^; passive smoker: 1335.1 ± 487.1 cm^2^; active smoker: 1513.1 ± 592.3 cm^2^; p=0.123). PMI also did not differ significantly across groups (non-smoker: 4.628 (IQR: 3.868–5.426); passive smoker: 4.407 (IQR: 3.511–5.591); active smoker: 4.888 (IQR: 3.776–6.253) cm^2^/m^2^; p=0.138) (Table 2). To assess the independent association between smoking status and muscle mass after adjusting for potential confounders, multivariate linear regression was performed with PMI as the continuous outcome. After adjustment for BMI, sex, and age (R^2^=0.451), neither passive smoking (β= -0.034; 95%CI: -0.395–0.328, p=0.855) nor active smoking (β=0.263; 95% CI: -0.101–0.627, p=0.158) was significantly associated with PMI. BMI (β=0.135; p<0.001), sex (β=1.658, p<0.001), and age (β= -0.037, p<0.001) were the only significant independent predictors of PMI. Multivariate logistic regression with CT-defined low psoas muscle mass as the binary outcome confirmed these findings. After adjustment, neither passive smoking (AOR=1.934; 95% CI: 0.899–4.158, p=0.091) nor active smoking (AOR=0.752; 95% CI: 0.322–1.758, p=0.511) was significantly associated with sarcopenia risk. Age (AOR=1.042; 95% CI: 1.009–1.077, p=0.012) and BMI (AOR=0.837; 95% CI: 0.754–0.930, p=0.001) were the only significant independent predictors of CT-defined low psoas muscle mass (Table 3). Subgroup analyses were performed to evaluate the robustness of the null finding. Sex-stratified analysis revealed no significant difference in PMI across smoking groups in either males (non-smoker: 5.559 ± 1.435; passive: 5.564 ± 1.492; active: 5.827 ± 1.746 cm^2^/m^2^; p=0.633) or females (non-smoker: 4.066 ± 0.979; passive: 3.681 ± 0.836; active: 3.736 ± 0.593 cm^2^/m^2^; p=0.114). BMI-stratified analysis showed no significant difference in PMI across smoking groups in either the BMI <25 group (p=0.463) or the BMI ≥25 (kg/m^2^) group (p=0.335). Age-stratified analysis revealed a significant difference in PMI across smoking groups among participants under 45 years of age (n=148; p=0.006), with active smokers demonstrating numerically higher PMI (median 5.355 cm^2^/m^2^) compared to passive smokers (4.348 cm^2^/m^2^) and non-smokers (4.638 cm^2^/m^2^). No significant difference was found among participants aged ≥45 years (n=113; p=0.672). These stratified findings suggest that age may act as an important effect modifier in the relationship between smoking and psoas muscle mass.

**Table 2:** Psoas muscle measurements by smoking status, Akdeniz University Hospital, Antalya, Turkey, 2019–2022 (N=261)

	*Passive smokers*	*Active smokers*	*Non-smokers*	*Total*	*p*
Albumin (g/L)	45 (43–46)	44 (42–46)	44 (42–47)	45	0.408^a^
CRP (mg/L)	1.7 (1–3)	2 (1–3.9)	1.5 (1–2.7)	2	0.285^a^
ALT (U/L)	20 (13–24)	19 (14–24)	20 (15–24)	20	0.435^a^
AST (U/L)	18 (16–23)	19 (15–23)	19 (16–22)	19	0.772^a^
GFR (mL/min/1.73m²)	105.0 ± 11.7	102.6 ± 11.5	103.1 ± 12.0	103.59	0.523^b^
Hemoglobin (g/dL)	13.7 (12.8–14.7)	14.4 (13–15.3)	13.5 (12.9–14.6)	13.9	0.029^a^
TPA (cm^2^)	1259 (926–1697)	1425 (1022–1873)	1267 (1034–1656)	1292	0.123^a^
PMI (cm^2^/m^2^)	4.41 (3.47–5.62)	4.89 (3.72–6.31)	4.63 (3.86–5.43)	4.62	0.138^a^

Values presented as median (IQR) or mean ± SD. P-values refer to Kruskal-Wallis between-group comparisons. Statistical significance threshold: p<0.05.

a Kruskal-Wallis H test, *post hoc* Bonferroni correction.

b One-way ANOVA.

**Table 3:** Multivariate linear and logistic regression analyses for predictors of Psoas muscle mass, Akdeniz University Hospital, Antalya, Turkey, 2019–2022 (N=261)

*Variable*	*Linear regression β (95% CI)*	*p*	*Logistic regression AOR (95% CI)*	*p*
Passive vs non-smoker	-0.034 (-0.395–0.328)	0.855	1.934 (0.899–4.158)	0.091
Active vs non-smoker	0.263 (-0.101–0.627)	0.158	0.752 (0.322–1.758)	0.511
BMI (kg/m^2^)	0.135 (0.096–0.175)	<0.001	0.837 (0.754–0.930)	0.001
Sex (male)	1.658 (1.354–1.961)	<0.001	1.335 (0.687–2.595)	0.393
Age (years)	-0.037 (-0.052 – -0.022)	<0.001	1.042 (1.009–1.077)	0.012
R^2^	0.451			

AOR: adjusted odds ratio. Reference category for smoking status: Non-smoker. Statistical significance threshold: p<0.05.

## DISCUSSION

We investigated the association between cigarette smoking exposure and CT-defined low psoas muscle mass using the Psoas Muscle Index (PMI) in 261 adult participants categorized as non-smokers, passive smokers, and active smokers. Our results demonstrated no statistically significant difference in PMI values across the three smoking groups (p=0.138), and this null finding remained robust after adjustment for BMI, sex, and age in multivariate linear regression (R^2^=0.451) and logistic regression analyses. Furthermore, sex-, age-, and BMI-stratified subgroup analyses confirmed the absence of a significant association in most subgroups, supporting the reliability of our primary finding.

Our null findings appear to contrast with several large-scale prospective studies demonstrating a significant association between smoking and sarcopenia risk. Several prospective cohort studies have reported that smokers experienced significantly greater muscle mass loss over follow-up compared to non-smokers, and that cumulative smoking exposure measured in pack-years was independently associated with lower skeletal muscle mass index^17,18^. The discrepancy between our findings and prior literature may be explained by several factors. First, the absence of pack-years data in our study precluded a dose-response analysis, and it is possible that only heavy or long-term smokers demonstrate measurable muscle mass reduction detectable at the population level. Second, our cohort consisted of relatively young adults (mean age 44.7 years) attending a tertiary hospital for routine clinical indications, which may have introduced selection bias toward healthier, more physically active individuals. Third, the use of PMI – which reflects psoas muscle mass specifically rather than total skeletal muscle mass – may not fully capture the systemic muscle wasting associated with chronic smoking exposure.

The significantly higher BMI observed in non-smokers compared to both passive and active smokers (p=0.017) in our study is consistent with the well-documented inverse relationship between smoking and body weight. Smoking is known to suppress appetite and increase basal metabolic rate through nicotine-mediated mechanisms, resulting in lower body weight and BMI among smokers. Despite this BMI difference between groups, our multivariate regression demonstrated that the null association between smoking status and PMI remained robust after BMI adjustment, suggesting that BMI differences between groups do not account for the null finding and that smoking status exerts no independent effect on psoas muscle mass in our cohort.

The biological mechanisms by which cigarette smoking is hypothesized to promote muscle loss are multifaceted. Tobacco smoke generates reactive oxygen species (ROS) that promote systemic oxidative stress, accelerating protein degradation and impairing muscle protein synthesis. Nicotine suppresses insulin-like growth factor-1 (IGF-1) signaling, a key anabolic pathway for skeletal muscle maintenance. Chronic smoking also elevates pro-inflammatory cytokines, including TNF-α and IL-6, which activate the ubiquitin-proteasome pathway and promote muscle atrophy. Additionally, smoking-induced hypoxia may impair mitochondrial function and reduce the oxidative capacity of skeletal muscle fibers. Despite these well-established mechanistic pathways, our study did not detect a significant association between smoking status and PMI, suggesting that these mechanisms may require longer exposure durations, higher cumulative doses, or specific populations (such as older adults or those with comorbidities) to produce measurable effects on psoas muscle mass^15^.

A key methodological consideration of this study is the use of population-derived sex-specific PMI cutoff values rather than externally validated thresholds. This approach was necessitated by the absence of universally accepted CT-based PMI thresholds for the Turkish adult population and the incompatibility of DXA- or BIA-based international criteria (EWGSOP2, AWGS2) with our CT-derived dataset. The lowest quintile approach, as originally described by Mourtzakis et al.^16^, is a well-established methodology in CT-based body composition research and has been applied across numerous published studies in various clinical settings. The resulting cutoffs (males: 4.421 cm^2^/m^2^, 95% CI: 4.179–4.621; females: 3.150 cm^2^/m^2^, 95% CI: 3.082–3.261) with bootstrap-derived confidence intervals provide a transparent and reproducible threshold definition appropriate for this exploratory study in a Turkish adult population. We acknowledge that these thresholds were not validated against EWGSOP2 or AWGS2 criteria, which additionally require assessment of muscle strength and physical performance – measurements unavailable in this retrospective dataset.

An interesting finding emerged from our age-stratified subgroup analysis. Among participants aged <45 years (n=148), a statistically significant difference in PMI was observed across smoking groups (p=0.006), with active smokers demonstrating numerically higher PMI compared to passive and non-smokers. This paradoxical finding may reflect the healthy worker effect – whereby younger active smokers in this hospital-based cohort may have been more physically active or engaged in manual labor, which is known to preserve or increase muscle mass. Alternatively, younger smokers may not yet have accumulated sufficient cumulative exposure to produce detectable muscle loss. This age-dependent pattern highlights the complexity of the smoking-muscle mass relationship and suggests that age may serve as an important effect modifier, warranting further investigation in dedicated prospective studies with age-stratified designs.

Regarding the generalizability of our findings, this study was conducted in a single tertiary care center in Antalya, Turkey, and the results may not be directly applicable to other ethnic groups, geographical regions, or clinical settings. The hospital-based recruitment strategy may have introduced selection bias, as participants who underwent CT for routine clinical indications may represent a healthier subset of the general population. Future multicenter studies incorporating community-based sampling are needed to confirm the external validity of these findings.

### Limitations

This study has several limitations that should be considered when interpreting the findings. First, as a cross-sectional secondary data analysis, causal inference between smoking exposure and psoas muscle mass cannot be established. Second, the retrospective design and hospital-based recruitment may have introduced bias, as only patients who had undergone CT imaging for clinical indications were included, which may not represent the general population. Third, PMI measurements were performed by a single radiologist who was not blinded to smoking status, which may have introduced observer and measurement bias; future studies should incorporate inter-rater reliability assessment. Fourth, despite adjustment for BMI, sex, and age, residual confounding from unmeasured variables – including physical activity level, dietary protein intake, vitamin D status, and hormonal factors – cannot be excluded^21^. Fifth, the absence of pack-years data, smoking duration, and cessation length precluded a dose-response analysis, which represents a significant limitation in characterizing the relationship between cumulative smoking exposure and muscle mass. Sixth, the passive smoking classification relied on self-report without objective biomarker confirmation (e.g. cotinine measurement), which may have introduced exposure misclassification. Seventh, PMI reflects psoas muscle mass at a single anatomical level (L3) and is not recognized as a standalone sarcopenia diagnostic tool by EWGSOP2 or AWGS2 guidelines, which additionally require assessment of muscle strength and physical performance.

### Future research

Our findings suggest that, in this group of otherwise healthy Turkish adults, smoking status was not independently associated with CT-defined low psoas muscle mass after adjustment for key confounders. These results should be interpreted in the context of the study’s limitations and do not preclude a potential association in other populations or with more detailed smoking exposure characterization. Future studies should involve larger and more diverse cohorts to comprehensively assess the effects of smoking on sarcopenia. Longitudinal studies are especially important for determining the long-term impact of smoking on muscle mass. Evaluating other muscle groups and their relationship with smoke exposure would also provide further insight.

Another research priority is to explore the underlying biochemical mechanisms by which smoke exposure may contribute to sarcopenia. The effects of tobacco smoke constituents on muscle protein synthesis and degradation – as well as on intramuscular inflammation – should be investigated further.

## CONCLUSIONS

This secondary data analysis found no statistically significant association between cigarette smoking exposure – whether passive or active – and CT-defined low psoas muscle mass as assessed by PMI in otherwise healthy Turkish adults. These findings were robust across sex-, age-, and BMI-stratified subgroup analyses and remained non-significant after adjustment for key confounders, including BMI, sex, and age. However, an age-stratified analysis revealed a significant difference in PMI among participants under 45 years, suggesting that age may moderate the relationship between smoking and muscle mass. Given the study’s limitations, including the absence of pack-years data and cross-sectional design, these findings should be interpreted cautiously. Prospective studies incorporating detailed quantitative smoking exposure data, objective muscle strength and physical performance assessments, and community-based sampling are needed to fully elucidate the relationship between tobacco exposure and skeletal muscle mass across different populations and age groups.

## Supplementary Material



## Data Availability

The data supporting this research are available from the authors on reasonable request.
